# GAP Score and CA-153 Associated with One-Year Mortality in Anti-MDA-5 Antibody-Positive Patients: A Real-World Experience

**DOI:** 10.3390/jcm10225241

**Published:** 2021-11-11

**Authors:** Chih-Wei Tseng, Kao-Lun Wang, Pin-Kuei Fu, Cheng-Yi Huang, Tsu-Yi Hsieh, Chia-Wei Hsieh, Kuo-Lung Lai, Wei-Ting Hung, Ching-Tsai Lin, Kuo-Tung Tang, Yi-Ming Chen, Wen-Nan Huang, Yi-Hsing Chen

**Affiliations:** 1 Division of Allergy, Immunology and Rheumatology, Department of Internal Medicine, Taichung Veterans General Hospital, Taichung 40705, Taiwan; cwtseng@vghtc.gov.tw (C.-W.T.); zuyihsieh@gmail.com (T.-Y.H.); chiaweih@gmail.com (C.-W.H.); kllaichiayi@yahoo.com.tw (K.-L.L.); wthung@vghtc.gov.tw (W.-T.H.); chingtsia@yahoo.com.tw (C.-T.L.); crashbug1982@gmail.com (K.-T.T.); gtim5555@yahoo.com (W.-N.H.); ysanne@vghtc.gov.tw (Y.-H.C.); 2 Department of Radiology, Taichung Veterans General Hospital, Taichung 40705, Taiwan; y0107124@yahoo.com.tw (K.-L.W.); antidonor@gmail.com (C.-Y.H.); 3 Department of Critical Care Medicine, Taichung Veterans General Hospital, Taichung 40705, Taiwan; yetquen@gmail.com; 4 College of Human Science and Social Innovation, HungKuang University, Taichung 43302, Taiwan; 5 Integrated Care Center of Interstitial Lung Disease, Taichung Veterans General Hospital, Taichung 40705, Taiwan; 6 Department of Medical Education, Taichung Veterans General Hospital, Taichung 40705, Taiwan; 7 Ph.D. Programme of Business, Feng Chia University, Taichung 40724, Taiwan; 8 Rong Hsing Research Center for Translational Medicine & Ph.D. Program in Translational Medicine, National Chung Hsing University, Taichung 40227, Taiwan; 9 School of Medicine, College of Medicine, National Yang Ming Chiao Tung University, Taipei 11221, Taiwan; 10 College of Medicine, National Chung Hsing University, Taichung 40227, Taiwan; 11 Department of Medical Research, Taichung Veterans General Hospital, Taichung 40705, Taiwan

**Keywords:** GAP score, CA-153, anti-MDA-5, idiopathic inflammatory myositis, interstitial lung disease, mortality

## Abstract

Background. Anti-melanoma differentiation-associated gene 5 (MDA-5) antibody is associated with respiratory failure and death in patients with idiopathic inflammatory myositis (IIM) and interstitial lung disease (ILD). This study aimed to investigate clinical parameters associated with mortality in anti-MDA-5 antibody-positive patients. Methods. We retrospectively reviewed the clinical and laboratory data, and pulmonary function test results in 55 anti-MDA-5 antibody-positive patients. A comparison was made between the survivors and non-survivors at the 12-month follow-up. Results. A total of 13 patients (23.6%) died within 12 months. Non-survivors had higher GAP scores (gender, age, and physiology score for idiopathic pulmonary fibrosis) (1 vs. 6, *p* < 0.01) and CA-153 (16.4 vs. 72.9, *p* < 0.01). In addition, rapid progressive ILD, fever, peak ferritin, leukocyte count, lactate dehydrogenase, CT score, intravenous immunoglobulin, mycophenolic acid, CMV infections, pneumocystis pneumonia, and pneumothorax were significantly associated with increased risks of 1-year mortality, while forced vital capacity, forced expiratory volume in one second, and diffusion capacity for carbon monoxide were correlated with decreased risk of 1-year mortality. Conclusions. Our study results suggest that GAP scores and CA-153 could be prognostic factors for 1-year mortality in anti-MDA-5 antibody-positive patients. A prompt pulmonary function test and CA-153 are essential for these patients to guide further management.

## 1. Introduction

Anti-melanoma differentiation-associated gene 5 (MDA-5) antibody (Ab) is a myositis-specific antibody (MSA) associated with poor prognosis in patients with rapid progressive interstitial lung disease (RP-ILD) [1]. It was recognized in 2005 and originally described as anti-CADM-140 in patients with amyopathic dermatomyositis [2]. Anti-MDA-5 positivity is known to be a poor prognostic factor [3]. Patients could have complications, such as pneumothorax and pneumomediastinum [4,5]. The presence of anti-MDA-5 could lead to a devastating condition, including respiratory failure and death. It can be found in patients with idiopathic inflammatory myositis (IIM) or patients with interstitial lung disease (ILD). Anti-MDA-5 antibody is one of the autoimmune features described in interstitial pneumonia with autoimmune features (IPAF). It was reported that IPAF patients, like patients with connective tissue disease related interstitial lung disease (CTD-ILD), should be closely monitored and put on early immunosuppressive treatment, because IPAF and CTD-ILD share similar CT findings [6].

The GAP score (abbreviation for gender, age, physiology) is a scoring method composed of age, gender, and pulmonary function results, and is widely used in patients with idiopathic pulmonary fibrosis (IPF) [7]. GAP scores predicted mortality in IPF patients [8]. This scoring system can be divided into three stages (I-III) and is associated with 1-year mortality of 6, 16, and 39%, respectively. An ILD-GAP model was developed to better predict mortality across all chronic ILD subtypes including CTD [9]. It was shown that the GAP model performed well in patients with rheumatoid arthritis complicated with ILD [10]. However, conflicting results existed. One study stated that the ILD-GAP model performed poorly in Jo-1-positive myositis-associated ILD [11]. Another study showed that the ILD-GAP model performed well for risk prediction of mortality among patients with IIM and acute exacerbation of ILD [12]. It remains unclear whether the GAP score is associated with poor outcome in IIM patients with ILD.

CA-153 or cancer antigen 15-3 has been used primarily in patients with breast cancer follow-up. Our previous study found no association between raised tumor markers and malignancy in patients with IIM [13]. Yet, we noted that CA-153 was associated with ILD [13]. A previous study also showed that CA-153 was highly associated with KL-6 in patients with sarcoidosis [14]. However, whether CA-153 is of prognostic value in patients with positive anti-MDA-5 antibody remains unknown.

Patients with anti-MDA-5 antibody have higher risks of pulmonary manifestations and complications with increased mortality. It is critical to identify factors related to survival once the presence of anti-MDA-5 has been detected. To our knowledge, no study has investigated the GAP model and CA-153 in patients with anti-MDA-5-associated diseases. The aim of the study was to assess whether the GAP model and clinical parameters including CA-153 predict mortality in anti-MDA-5-positive patients.

## 2. Materials and Methods

### 2.1. Patients

In total, 71 consecutive anti-MDA-5-positive patients were identified when anti-MDA-5 tests were available in the hospital. Medical records of their first hospital visits between March 2017 and May 2021 were reviewed. Three patients who were under the age of 20 were excluded. Thirteen patients who refused to participate in the study were excluded. A final of 55 anti-MDA-5 patients joined the study.

The diagnosis of IIM was made according to the 2017 EULAR/ACR criteria for idiopathic inflammatory myositis [15]. IPAF was defined according to an official research statement by European Respiratory Society/American Thoracic Society [16].

All clinical and laboratory data were collected from the first visit to the hospital to the one-year follow-up or to the final observation day (20 May 2021). Diagnosis of interstitial lung disease was made according to HRCT or lung biopsy after multi-disciplinary discussion. Written informed consent was obtained including permission for retrospective analysis of their symptoms/signs, laboratory data, and CT images upon the first visit to the hospital. The study was approved by Institutional Review Board of Taichung Veterans General Hospital (CE17037B).

### 2.2. Mortality Outcome

Survival status and causes of death of these 55 patients between the first visit at the hospital and the 12-month follow-up were obtained from both inpatient and outpatient medical records.

### 2.3. Identification of Clinical Symptoms, Comorbidities, and Complications

Clinical manifestations at the first visit including fever, Gottron’s sign, and other skin rashes were collected by experienced rheumatologists. Moreover, histories of smoking, diabetes mellitus, and hypertension were also recorded. Complications including CMV infection, pneumocystis pneumonia (PCP) infection, pneumomediastinum, and pneumothorax were recorded. RP-ILD was defined by the presence of deterioration of dyspnea, with a decrease in the partial pressure of oxygen levels or emerging radiographic anomalies in the past 4 weeks without evidence of infection [17].

### 2.4. Laboratory Evaluation

Laboratory evaluation included initial complete blood count, creatine phosphokinase (CK), and lactate dehydrogenase (LDH).

Anti-MDA-5 and anti-Ro-52 were determined by Euroimmun myositis Line Immunoassays (LIA). A EUROLINE Autoimmune Inflammatory Myopathies 16 Ag profile test was carried out in an automatic processing EUROBlotOne machine (Euroimmun, Lübeck, Germany), which has been available in the hospital since March 2017.

Cancer antigen 15-3/CA-153 was tested using electrochemiluminescence immunoassay in cobas e602 (Roche, Basel, Switzerland). The initial CA-153 value was recorded during the 12-month follow-up.

Peak ferritin values during the follow-up period were analyzed with a Siemens Healthcare Diagnostics (Erlangen, Germany) Centaur XP using chemiluminescence (CLIA).

### 2.5. GAP Score

Components of the GAP score are shown in Supplementary Materials Table S1. By the addition of assigned points of gender, age, predicted forced vital capacity (FVC), and predicted diffusion capacity (DLco), a GAP score ranging between 0 and 8 was obtained. Then, the patients were categorized into three groups based on the GAP score: stages I (0–3), II (4–5), and III (6–8). The first pulmonary function test results were recorded upon the first visit to the hospital.

Only 43 patients had initial pulmonary function test (PFT) results and their GAP scores were calculated based on their age at the first visit and PFT results. Eight patients failed to perform pulmonary function at the first visit due to respiratory distress. Their predicted FVC results in the GAP index were deemed to be <50%, which scored two in the calculation. The predicted DLco results were thus scored as three in the GAP calculation. The remaining four patients who did not have pulmonary symptoms and had unremarkable chest plain films did not have initial pulmonary function tests and their first pulmonary function test results during follow-up were unremarkable. These four patients had no ILD at the first-year follow-up. Their GAP scores in the pulmonary function domain were designated as zero.

### 2.6. HRCT Scoring

**Figure 1:**
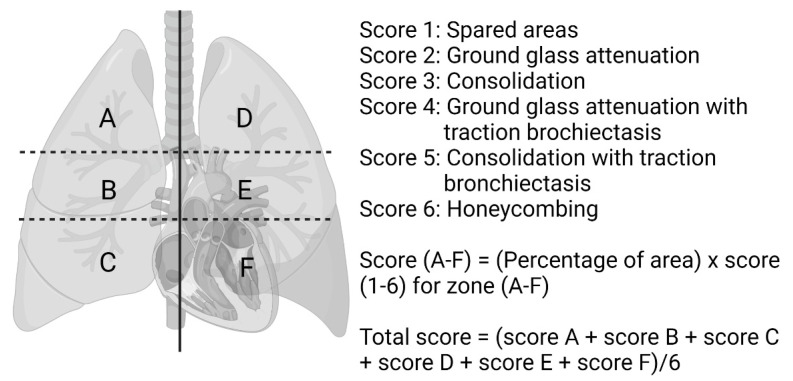
CT Scoring method [20].

The initial chest computed tomography and high-resolution CT images were reviewed. The images were reviewed by one rheumatologist and one radiologist for the presence, extent, and distribution of CT findings, and the findings were agreed upon by two radiologists. The presence and pattern of ILD was approached using the American thoracic society consensus criteria for idiopathic interstitial pneumonias [18]. If no agreement was met, then the images were reviewed in the multidisciplinary discussion (MDD), which was held monthly by pulmonologists, radiologists, rheumatologists, pathologists, and cardiologists. Patterns of usual interstitial pneumonia were made according to the official practice guideline by Raghu G. et al. in 2018 [19]. The CT findings were graded on a 6-score scale according to the Ichikado ARDS score [20], which has been adopted in a prior study of patients with anti-MDA-5 antibody-positive clinically amyopathic dermatomyositis [5]. The lungs were divided into six zones and each zone was graded separately. The upper zone for each lung was defined as above the level of the carina and the middle zone as the area between the carina and the inferior pulmonary vein, and the lower zone below the inferior pulmonary vein. The abnormal findings and extent were evaluated visually and estimated to the nearest 10% of the parenchymal involvement. For each zone, CT findings were graded as follows: score of 1 for spared areas; score of 2 for ground glass attenuation; score of 3 for consolidation; score of 4 for ground glass attenuation with traction bronchiectasis or bronchiolectasis; score of 5 for consolidation with traction bronchiectasis or bronchiolectasis; and score of 6 for honeycombing. For each zone, a score was calculated by multiplying the percentage area by the value score 1–6. The overall CT score was determined by adding the scores in six zones and dividing by six (Figure 1). Six patients did not have the initial CT available as the patients had no diagnosis of ILD.

### 2.7. Pharmacologic Therapy

During the 12-month follow up, treatments of methylprednisolone pulse therapy, cyclophosphamide, intravenous immunoglobulin, mycophenolic acid derivatives (MPA) (Myfortic or Cellcept), rituximab, azathioprine, calcineurin inhibitor (cyclosporin or tacrolimus), Janus kinase inhibitors, and anti-fibrotic agents were recorded.

### 2.8. Statistical Analysis

The continuous variables were expressed as medians and range; categorical variables were expressed as numbers followed by a percentage. The differences between the survivors and non-survivors were compared using the Mann–Whitney U test or Fisher’s Exact test. The Cox proportional hazards regression models were used to identify independent predictors of mortality. Variables identified as significant in the univariate analysis were further adjusted by age at first visit and sex in the multivariate analysis. Statistical significance was set at *p* < 0.05. Statistical analyses were carried out by IBM SPSS Statistics for Windows, version 22(IBM Corp., Armonk, NY, USA). Receiver operating characteristic (ROC) curves were drawn for significant continuous variables to determine the best-cut-off values for the prediction of mortality using Youden’s index. Comparisons of patient survival according to different GAP stages and CA-153 as well as the CT score and ferritin cut-off value were conducted using Kaplan–Meier survival curves. The log-rank test was used (MedCalc version 19.6, Ostend, Belgium).

## 3. Results

### 3.1. Demographic Characteristics

**Figure 2:**
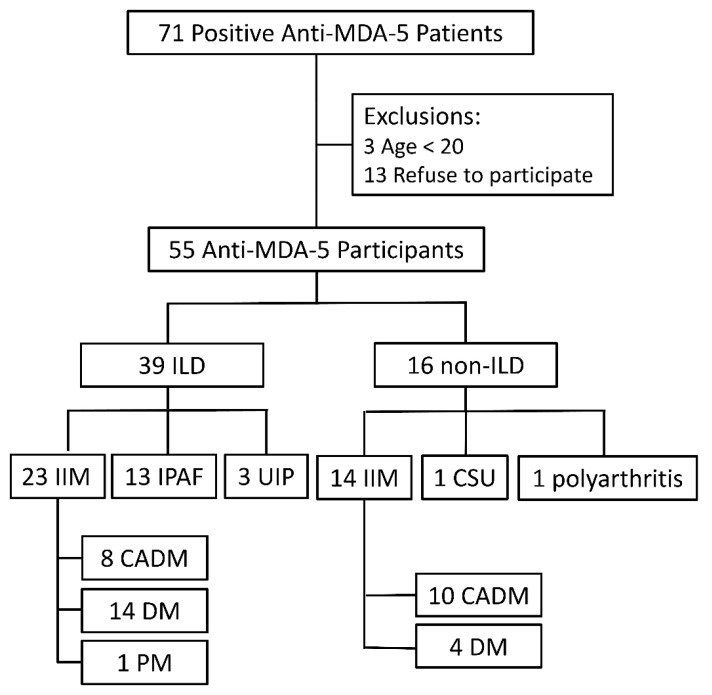
Patient classification by the presence of ILD and IIM diagnosis.

Among 55 participants with positive anti-MDA-5 antibodies (Figure 2), 39 patients (70.9%) had been diagnosed with ILD by the final observation day. Among 39 ILD patients, 23 met the idiopathic inflammatory myositis (IIM) diagnosis, and the other 15 patients were classified as IPAF. In subjects with ILD, 30 (76.9%) were classified as organizing pneumonia without non-specific interstitial pneumonia (NSIP), 5 (12.8%) had organizing pneumonia overlapping with NSIP, 1 NSIP, and 3 usual interstitial pneumonia (UIP). Among 37 IIM patients, 18 were categorized as clinically amyopathic dermatomyositis (CADM), 18 dermatomyositis, and 1 polymyositis. In patients without ILD, we recruited 14 IIM, 1 polyarthritis, and 1 chronic spontaneous urticaria.

### 3.2. Comparison of Survivors and Non-Survivors

Table 1 shows the clinical characteristics, pulmonary function test results, laboratory tests, and treatments between survivors and non-survivors.

A total of 13 patients (23.6%) died at a median age of 59.9 years within 1 year. Age, female sex, IIM diagnosis, diabetes mellitus, hypertension, Gottron’s sign, and CK were indistinguishable between survivors and non-survivors. Non-survival was associated with smoking, RP-ILD, fever, elevated peak ferritin and CA-153, elevated WBC, elevated LDH, higher GAP scores and CT scores, treatments with mycophenolate and IVIG, and complications including CMV infection, PCP infection, pneumomediastinum (PM), and pneumothorax (PTX). The values of FEV1/FVC/DLco were higher in survivors compared with non-survivors.

### 3.3. Predictors of Mortality

**Figure 3:**
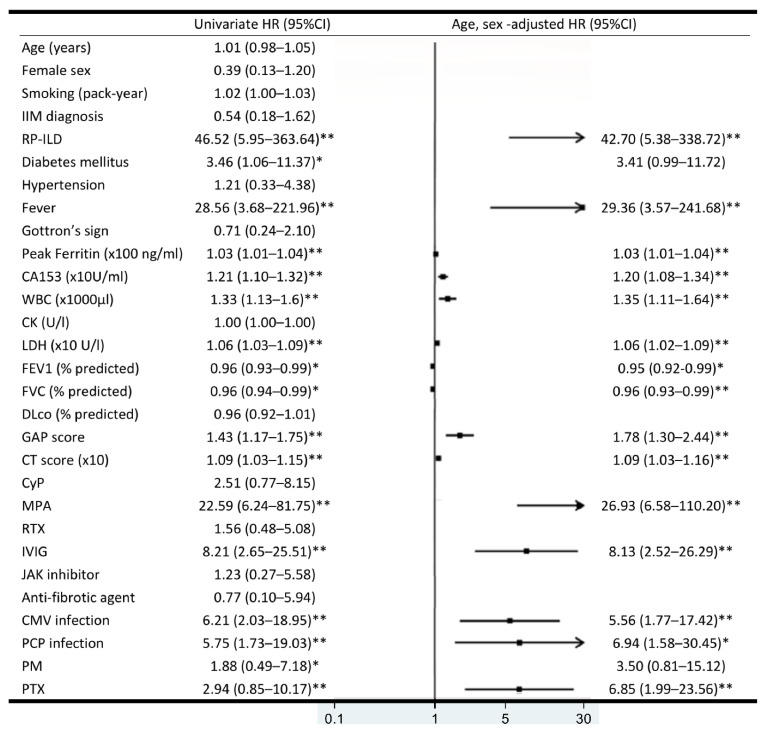
Univariate and age- and sex-adjusted hazard ratio (HR) for mortality. HR plotted in the middle; the 95% confidence intervals presented in parentheses. * *p* < 0.05, ** *p* < 0.01.

Cox proportional hazard regression analyses for 1-year survival are shown in Figure 3. In the age- and sex-adjusted analysis, RP-ILD (HR 42.70, 95% CI 5.38–338.72), fever (HR 29.36, 95% CI 3.57–241.68), peak ferritin (HR 1.03, 95% CI 1.01–1.04), CA-153 (HR 1.20, 95% CI 1.08–1.34), WBC (HR 1.35, 95% CI 1.11–1.64), LDH (HR 1.06, 95% CI 1.02–1.09), GAP score (HR 1.78, 95% CI 1.30–2.44), CT score (HR 1.09, 95% CI 1.03–1.16), IVIG (HR 8.13, 95% CI 2.52–26.29), MPA (HR 26.93, 95% CI 6.58–110.20), CMV infection (HR 5.56, 95% CI 1.77–17.42), PCP infection (HR 6.94, 95% CI 1.58–30.45), and PTX (HR 6.85, 95% CI 1.99–23.56) were significantly correlated with increased risk of 1-year mortality. FEV1 (HR 0.95, 95% CI 0.92–0.99), FVC (HR 0.96, 95% CI 0.93–0.99), and DLco (HR 0.93, 95% CI 0.87–1.00) were associated with decreased risk of 1-year mortality. ROC analysis was used to determined cut-offs for the significant variables associated with 1-year mortality, including peak ferritin, CA-153, WBC, LDH, GAP score, and CT score (Supplementary Materials Table S2).

### 3.4. One-Year Survival Analysis

**Figure 4:**
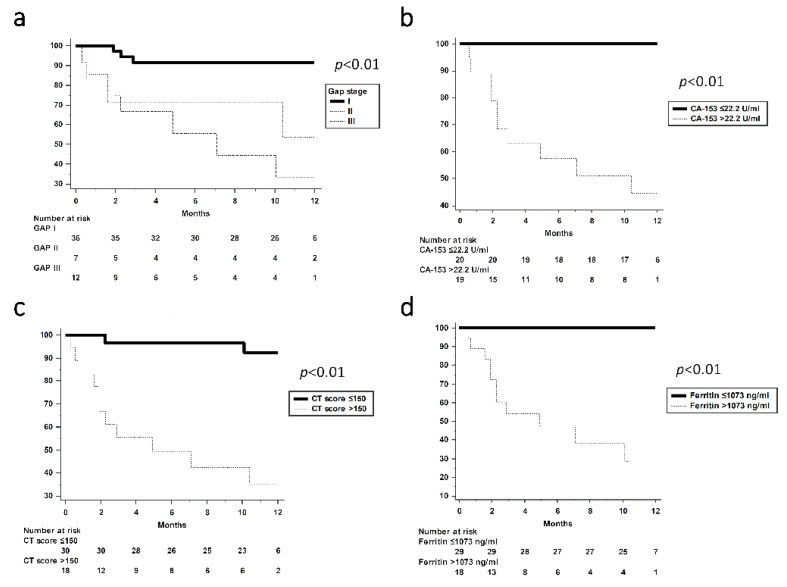
Survival analysis according to (**a**) GAP stage, (**b**) CA-153, (**c**) CT score, and (**d**) ferritin. *p* < 0.05 by the log-rank test. (**a**) GAP stage I vs. stage II, *p* < 0.01. (**a**) GAP stage I vs. stage III, *p* < 0.01. GAP—Gender, Age, and Physiology score for idiopathic pulmonary fibrosis.

One-year survival curves according to GAP stages, CA-153, CT scores, and peak ferritin levels are shown in Figure 4. All patients with stage I–III GAP had one-year mortality rates of 8.3%, 42.9%, and 58.3%, respectively (Figure 4a). Patients with GAP stage I appeared to have better survival than GAP stages II and III. Patients with CA-153 > 22.2 U/mL had an increased one-year mortality rate than those with CA-153 ≤ 22.2 U/mL (42.1% vs. 0%, *p* < 0.01, Figure 4b). In addition, participants with CT score >150 and ferritin level >1073 ng/mL were associated with poor survival (Figure 4c,d). In MDA-5-positive patients with ILD, GAP stage, CA-153, CT score, and ferritin were also associated with increased mortality (Supplementary Materials Figure S1).

### 3.5. Treatment and Causes of Death in Non-Survivors

Table 2 shows the details of treatments and causes of death in non-survivors. There were eight males and five females. All non-survivors except one had RP-ILD. Seven patients (53.8%) fulfilled the criteria for IIM diagnosis. Four patients (30.8%) died of ILD, and nine patients (69.2%) died of opportunistic infections. The leading cause of infections was CMV and PCP. Seven patients received MPA and five patients died of CMV infection, among whom four had concomitant PCP infection. Four patients received rituximab, but three patients died of opportunistic infections.

## 4. Discussion

This medical record review study investigated the clinical prognostic factors in our patients who tested positive for anti-MDA-5 autoantibodies. We reviewed 55 patients and 13 of them died within 1 year. Six patients (46.2%) died of CMV infection. We found that the GAP score and CA-153 could help predict 1-year mortality. Our results indicate that patients with anti-MDA-5-positive autoantibodies should receive pulmonary function tests at baseline soon after diagnosis for risk stratification. In cases of severe respiratory failure or pneumothorax when the pulmonary function test cannot be performed, the GAP score should be interpreted as stage III.

Studies have shown that the GAP score predicts 1-year mortality in IPF patients and rheumatoid arthritis patients [8,10]. No study to date has investigated GAP scores primarily in anti-MDA-5-positive patients. The present study is the first to demonstrate that the GAP score could also predict the 1-year survival of anti-MDA-5-positive patients. The GAP score is composed of gender, age, predicted FVC, and predicted DLco. Older age could also be associated with multiple comorbidities that are associated with mortality. We found that the GAP score, combining age and baseline pulmonary function test results, predicted the mortality of anti-MDA-5-positive patients. Rheumatologists should be aware of a higher 1-year mortality rate (>50%) in GAP stage 2 and 3 patients.

Previous studies showed that anti-MDA-5-positive dermatomyositis patients can be divided into chronic ILD and RP-ILD [21]. Elderly age at onset, poor oxygenation, and higher ferritin were associated with RP-ILD [22]. A higher ferritin level also predicted mortality [19,20]. In line with these studies, our results showed 18 RP-ILD and 21 chronic ILD at the 1-year follow-up. A higher ferritin level was associated with non-survival. Patterns of ILD in our patients comprised mainly OP (76.9%) and OP overlapping NSIP (12.8%), which was consistent with a previous study that OP was the most prevalent form in anti-MDA-5 patients while NSIP predominates in anti-tRNA synthetase syndrome [23]. Among the five patients with OP overlapping NSIP in our study, three patients (60%) developed RP-ILD. A previous cohort study of this overlapping form also reported an increased risk of disease progression [24]. Further studies in larger series are warranted to confirm our findings.

Krebs von den Lungen 6 (KL-6), a human MUC1 mucin protein, has been extensively used as a potential biomarker to evaluate the severity of CTD-ILD. Studies have shown that CA-153 recognizes the core protein of mucin1 [25]. Type II pneumocytes represent a primary cellular source of mucin 1 in the affected lungs of patients with ILD. The changes in serum KL-6 levels in ILD patients with IIM also showed a significant inverse correlation with changes in FEV1 and DLco [26]. Our result was the first to demonstrate a predictive value of CA-153 for mortality in MDA-5 patients. We speculated that the levels of CA-153 may reflect the severity of CTD-ILD. A prospective study is needed to evaluate the clinical application of CA-153 in MDA-5 patients.

Patients with positive anti-MDA-5 antibody are usually considered to have “anti-MDA5-positive syndrome” with different phenotypes and survivals. Anti-MDA5-positive patients typically present with amyopathic dermatomyositis with ILD that can be fatal with RP-ILD; some may behave like an anti-synthetase syndrome. However, different phenotypes of anti-MDA5-positive patients have been described. Yves et al. proposed a classification algorithm based on Raynaud’s syndrome, arthritis/arthralgia, and gender [27]. Three subgroups of anti-MDA5-positive syndrome included clinical manifestations of RP-ILD, rheumatic dermatomyositis, and vasculopathic dermatomyositis. However, there was no universal agreement on how to stratify anti-MDA5-positive patients. Future studies are needed to address this issue.

In the present study, we also noted that fever, WBC, LDH, and CT score were independent risk factors for increased mortality. Consistent with previous studies, higher CT scores were associated with poor survival [5,28,29]. Fever, WBC, and LDH have seldom been reported in the previous literature [5,30]. It is worth noting that 92.3% of the non-survivors in this study had fever. Most of the non-survivors died of opportunistic infections (69.2%). Among these opportunistic infections, CMV infection was the most common cause of death. One recent Japanese study showed that a 40.4% CMV reactivation rate was observed in 52 dermatomyositis patients, and immunosuppressants and diabetes mellitus were thought to be the culprits [31].

Our results showed that MPA use was associated with worse survival, which can be confounded by indication. Most of the non-survivors received MPA for RP-ILD. We believe that aggressive treatment with immunosuppressants might lead to CMV infections. It has been reported that IL-15 was significantly elevated in non-survivors, most of whom had CMV antigenemia [32]. IL-15 is a proinflammatory cytokine, secreted mainly by mononuclear cells following viral infection. IL-15 also plays an important role in NK cell development from common lymphoid progenitors [33]. Moreover, it was recently shown that effector CD8+ T cells are preferentially maintained by the cytokine IL-15 in the peripheral blood as well as in the bone marrow after CMV infection [34,35]. MPA derivatives were found to inhibit NK cell proliferation and function [36]. In addition, inhibition of IL-15 production by MPA could dampen the development of NK cells and their ability to combat the CMV virus [37]. CMV prophylaxis should be considered mandatory in anti-MDA-5-positive patients receiving MPA drugs.

This study had inherited limitations. First, anti-MDA-5-positive disease is a rare condition and the limited number of patients prevented additional multivariate analysis. However, anti-MDA-5 is not uncommon in Asian populations. Hence, our results may be of interest for rheumatologists in this region as a novel tool for mortality risk prediction. Second, the study had a retrospective design and some data were missing. Not all patients received the laboratory tests for ferritin and CA-153. Nonetheless, our results suggest that laboratory evaluation, including leukocyte count and LDH, could also be used as feasible alternatives for prognosis. Third, therapeutic options of immunosuppressive agents could be confounded by indication and quantitative anti-MDA-5 antibody levels were lacking. It was difficult to ascribe outcomes to any treatment intervention. Future studies are needed to determine whether follow-up anti-MDA-5 levels can predict mortality.

## 5. Conclusions

Anti-MDA-5-positive antibody was associated with a high mortality rate. Our data indicate a high GAP stage and CA-153 are related to one-year mortality. The pulmonary function test and CA-153 are crucial to mitigate mortality risks in anti-MDA-5-positive patients.

## Figures and Tables

**Table 1:** Comparison between survivors and non-survivors at 1-year follow up.

	Survivors (*n* = 42)	Non-Survivors (*n* = 13)	*p*-Value
Age (years)	54.2 (40.4–63.8)	59.9 (51.2–62.3)	0.39
Female sex, no. (%)	28 (66.7)	5 (38.5)	0.08
Smoking (pack-year)	0 (0.0–5.5)	20.0 (0.0–40.0)	<0.05
IIM diagnosis, no. (%)	30 (71.4)	7 (53.8)	0.31
RP-ILD, no. (%)	6 (14.3)	12 (92.3)	<0.01
Diabetes mellitus, no. (%)	4 (9.5)	4 (30.8)	0.08
Hypertension, no. (%)	8 (19.0)	3 (23.1)	0.71
Fever, no. (%)	10 (23.8)	12 (92.3)	<0.01
Gottron’s sign, no. (%)	27 (64.3)	7 (53.8)	0.53
Peak Ferritin (*n* = 47, ng/mL)	300.0 (88.0–809.5)	2585.5 (1629.0–9556.0)	<0.01
CA153 (*n* = 39, U/mL)	16.4 (11.7–27.8)	72.9 (33.1–139.4)	<0.01
WBC (×1000 μL)	5.7 (4.9–8.1)	9.4 (6.3–13.6)	<0.01
CK (*n* = 52, U/L)	95.0 (57.0–235.0)	17 (37.5–349.5)	0.35
LDH (*n* = 51, U/L)	273.5 (180.0–345.0)	433.0 (295.0–581.5)	<0.01
FEV1 (*n* = 43, % predicted)	88.0 (67.3–100.0)	69.0 (50.0–69.0)	<0.05
FVC (*n* = 43, % predicted)	87.0 (69.0–99.8)	62.0 (49.0–69.0)	<0.01
DLco (*n* = 35, % predicted)	84.0 (60.0–98.0)	57.0 (47.8–63.3)	<0.05
GAP score	1 (0–3)	6 (3–6)	<0.01
CT score (*n* = 48)	106.7 (100.0–150.0)	203.3 (155.0–279.2)	<0.01
Treatment, no. (%)			
CyP	5 (11.9)	4 (30.8)	0.19
MPA	0 (0)	7 (53.8)	<0.01
RTX	1 (2.4)	4 (30.8)	0.72
IVIG	2 (4.8)	6 (46.2)	<0.01
JAK inhibitor	5 (11.9)	2 (15.4)	0.66
Anti-fibrotic agent	4 (9.5)	1 (7.7)	1.00
Complications, no. (%)			
CMV infection	3 (7.1)	6 (46.2)	<0.01
PCP infection	1 (2.4)	4 (30.8)	<0.01.
PM	1 (2.4)	3 (23.1)	<0.05
PTX	1 (2.4)	5 (38.5)	<0.01

Continuous variables were expressed and compared using the median (interquartile range). Abbreviations: IIM = Idiopathic inflammatory myositis; RP-ILD = Rapid progressive interstitial lung disease; WBC = White blood cell; CK = Creatinine kinase; LDH = Lactate dehydrogenase; FEV1 = Forced expiratory volume in one second; FVC = Forced vital capacity; DLco = Diffusion capacity for carbon monoxide; GAP score = Gender, Age, and Physiology score for idiopathic pulmonary fibrosis; CyP = Cyclophosphamide; MPA = mycophenolic acid derivatives; RTX = Rituximab; IVIG = Intravenous immunoglobulin; JAK = Janus kinase; CMV = Cytomegalovirus; PCP = Pneumocystis Pneumonia; PM = Pneumomediastinum; PTX = Pneumothorax.

**Table 2:** Treatment and causes of death in non-survivors.

	Age	Sex	IIM	RP-ILD	WBC	LDH	Treatment	Causes of Death
1	50	F	Y	Y	4300	277	Cs + CyP + MPA + CNI + IVIG	CMV, PCP, HCoV-NL63
2	43	M	Y	Y	9410	397	Cs + CyP + MPA + CNI + IVIG	CMV, PCP
3	45	M	N	Y	14,200	850	Cs + MPA + CNI	CMV, PCP
4	50	F	Y	Y	6520	436	Cs + MPA + CNI + RTX + IVIG	ILD
5	66	F	Y	Y	12,910	430	Cs + CyP + RTX + IVIG	CMV, TB
6	58	M	Y	Y	9550	218	Cs + TOF	Pseudomonas aeruginosa, Staphylococcus aureus, Klebsiella pneumoniae
7	51	M	Y	Y	8010	593	Cs + MPA + IVIG	CMV, PCP
8	61	M	N	Y	8670	NA	Cs	ILD
9	62	F	Y	Y	3960	722	Cs + CyP	ILD
10	60	M	N	Y	15,980	349	Cs + MPA + CNI + RTX	C. tropicalis
11	62	F	N	Y	6010	437	Cs + MPA + RTX + IVIG	CMV, NTM
12	76	M	N	Y	11,070	547	Cs + TOF + PFD	ILD
13	60	M	N	N	14,690	152	Cs	NTM

Abbreviations: IIM = Idiopathic inflammatory myositis; RP-ILD = Rapid progressive interstitial lung disease; WBC = White blood cell (μL); LDH = Lactate dehydrogenase (U/L); Cs = Corticosteroids; RTX = Rituximab; CyP = Cyclophosphamide; MPA = Mycophenolic acid derivatives; CNI = Calcineurin inhibitor; IVIG = Intravenous immunoglobulin; TOF = Tofacitinib; PFD = Pirfenidone; CMV = Cytomegalovirus; PCP = Pneumocystis pneumonia; HCoV-NL63 = Human coronavirus NL63; TB = Tuberculosis; NTM = Nontuberculous mycobacteria.

## Data Availability

Data will be available upon reasonable request.

## Supplementary Materials

The following are available online at https://www.mdpi.com/article/10.3390/jcm10225241/s1. Figure S1: Kaplan–Meier survival curves plotted for (a) GAP stages, (b) CA-153, (c) CT score, and (d) ferritin in patients with anti-MDA-5 antibody-positive ILD patients. Table S1: Components of the GAP score. Table S2: Cut-off levels of significant variables associated with 1-year mortality determined by ROC curves.
