# Supplementary material for: GAP Score and CA-153 Associated with One-Year Mortality in Anti-MDA-5 Antibody-Positive Patients: A Real-World Experience
Source: J Clin Med. 2021 Nov 11;10(22):5241. doi: 10.3390/jcm10225241 (PMC8618032; doi:10.3390/jcm10225241)
Supplement: Supplementary file 1 [file jcm-10-05241-s001.zip › jcm-1442365-supplementary.pdf]

Supplementary Table S1. Components of GAP score

GAP Score	0	1	2	3
Gender	Female	Male		
Age, years	≤60	61-65	>65	
FVC % Predicted	>75	50-75	<50	
DLco % Predicted	>55	36-55	≤35	Unable to do

FVC: forced vital capacity; DLco: diffusion capacity for carbon monoxide;

GAP: Gender, Age, and Physiology score for idiopathic pulmonary fibrosis.

Supplementary Table S2. Cut-off levels of significant variables associated with 1-year mortality determined by ROC curves.

	CT score	Peak Ferritin (ng/ml)	GAP score	CA-153 (U/ml)	WBC (μl)	LDH (U/l)
AUC	0.830	0.902	0.829	0.917	0.744	0.772
Cut-off	150	1073	3	22.2	7990	345
P value	<0.01	<0.01	<0.01	<0.01	<0.01	<0.01

GAP: Gender, Age, and Physiology score for idiopathic pulmonary fibrosis.

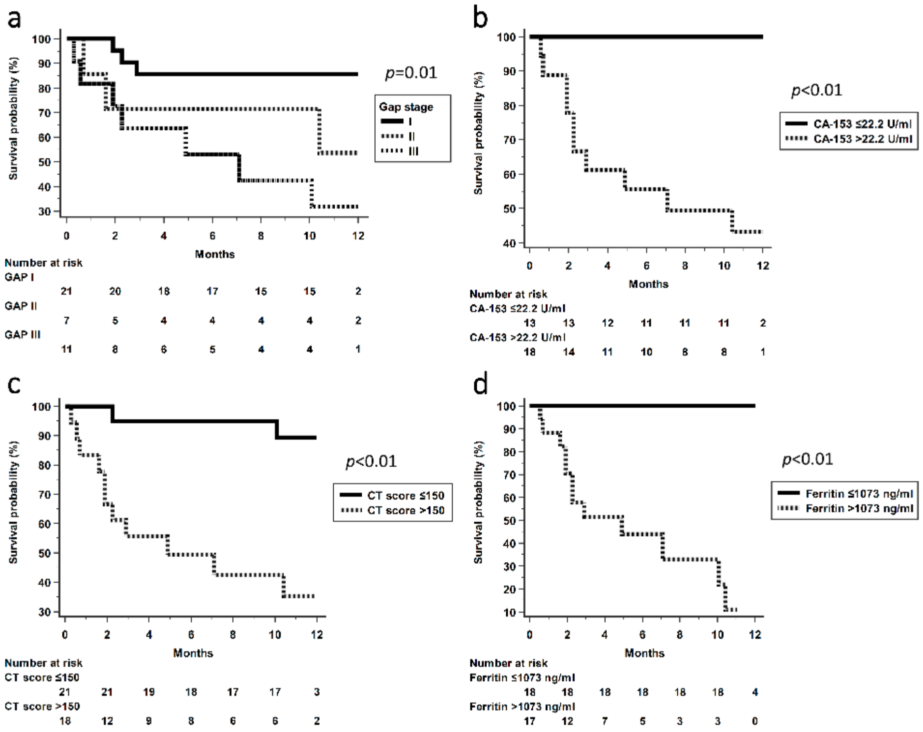

Supplementary Figure S1. Kaplan-Meier survival curves plotted for (**a**) GAP stages, (**b**) CA-153, (**c**) CT score, and (**d**) ferritin in patients with anti-MDA-5 antibody-positive ILD patients.
